# miR-125b modulates megakaryocyte maturation by targeting the cell-cycle inhibitor p19^INK4D^

**DOI:** 10.1038/cddis.2016.288

**Published:** 2016-10-20

**Authors:** Mingyi Qu, Fang Fang, Xiaojing Zou, Quan Zeng, Zeng Fan, Lin Chen, Wen Yue, Xiaoyan Xie, Xuetao Pei

**Affiliations:** 1 Stem Cell and Regenerative Medicine Lab, Beijing Institute of Transfusion Medicine, Beijing 100850, China; 2 South China Research Center for Stem Cell and Regenerative Medicine, South China Institute Biomedicine, Guangzhou 510005, China; 3 Southern Medical University, Guangzhou 510005, China

## Abstract

A better understanding of the mechanisms involved in megakaryocyte maturation will facilitate the generation of platelets *in vitro* and their clinical applications. A microRNA, miR-125b, has been suggested to have important roles in the self-renewal of megakaryocyte-erythroid progenitors and in platelet generation. However, miR-125b is also critical for hematopoietic stem cell self-renewal. Thus, the function of miR-125b and the complex signaling pathways regulating megakaryopoiesis remain to be elucidated. In this study, an attentive examination of the endogenous expression of miR-125b during megakaryocyte differentiation was performed. Accordingly, the differentiation of hematopoietic stem cells requires the downregulation of miR-125b, whereas megakaryocyte determination and maturation synchronize with miR-125b accumulation. The overexpression of miR-125b improves megakaryocytic differentiation of K562 and UT-7 cells. Furthermore, stage-specific overexpression of miR-125b in primary cells demonstrates that miR-125b mediates an enhancement of megakaryocytic differentiation after megakaryocyte determination, the stage at which megakaryocytes are negative for the expression of the hematopoietic progenitor marker CD34. The identification of miR-125b targets during megakaryopoiesis was focused on negative regulators of cell cycle because the transition of the G1/S phase has been associated with megakaryocyte polyploidization. Real-time PCR, western blot and luciferase reporter assay reveal that p19^INK4D^ is a direct target of miR-125b. P19^INK4D^ knockdown using small interfering RNA (siRNA) in megakaryocyte-induced K562 cells, UT-7 cells and CD61^+^ promegakaryocytes results in S-phase progression and increased polyploidy, as well as improved megakaryocyte differentiation, similarly to the effects of miR-125b overexpression. P19^INK4D^ overexpression reverses these effects, as indicated by reduced expression of megakaryocyte markers, G1-phase arrest and polyploidy decrease. P19^INK4D^ knockdown in miR-125b downregulated cells or p19^INK4D^ overexpression in miR-125b upregulated cells rescued the effect of miR-125b. Taken together, these findings suggest that miR-125b expression positively regulates megakaryocyte development since the initial phases of megakaryocyte determination, and p19^INK4D^ is one of the key mediators of miR-125b activity during the onset of megakaryocyte polyploidization.

Thrombocytopenia, the deficiency of platelets (PLTs) in the blood, threatens millions of people, including patients undergoing high-dose chemotherapy, and subjects affected by aplastic anemia or hepatitis virus-related cirrhosis. The cells responsible of PLT production are the megakaryocytes (MK). Polyploidization is an important step during MK maturation and PLT generation. To understand the mechanisms underlying MK maturation will facilitate *ex vivo* PLT manufacture for therapeutic applications and clinical treatments of thrombocytopenia.

MicroRNAs (miRNAs) are small non-coding RNA molecules that regulate gene expression primarily by inhibiting the translation of target mRNAs through direct binding of specific sites in the 3′-untranslated region (3′-UTR).^1^ It is now commonly accepted that miRNAs has essential roles in hematopoiesis, including embryonic stem cell differentiation, erythropoiesis, granulocytopoiesis/monocytopoiesis, lymphopoiesis and megakaryocytopoiesis.^2^ During MK determination and maturation, miR-155 blocks megakaryocytic differentiation by targeting Ets-1 and Meis1 transcription factors, miR-150 drives MK-erythroid precursors toward the megakaryocytic fate via the inhibition of the target transcription factor c-Myb and miR-34a enhances MK differentiation in hematopoietic stem cells (HSCs) through the repression of c-Myb and MEK1 expression.^3^

Recently, Klusmann *et al.*^4^ reported that the overexpression of the onco-miR miR-125b-2 could induce the proliferation and differentiation of MK and MK-erythroid precursors. The same authors also revealed that miR-125b enhanced the polyploidization and PLT output of MKs.^5^ Further, miR-125b-2 was found to have a direct correlation with the pathogenesis of acute megakaryoblastic leukemia in Down syndrome patients. Therefore, it is crucial to uncover the correlation between miR-125b and megakaryocytopoiesis. Although the study by Klusmann *et al.*^4^ suggests that Dicer and ST18 were direct targets of miR-125b in megakaryocytopoiesis, both of these factors are common to several cellular processes.^6^ Considering the significant effect of miR-125b on cell proliferation and endomitosis of MK progenitors and MKs, it is reasonable to speculate that miR-125b may regulate the expression of cell-cycle inhibitors. In this study, we systematically identified the role of miR-125b in the differentiation of MKs and one of the target genes that mediate its function, the cell-cycle inhibitor p19^INK4D^.

## Results

### Endogenous expression of miR-125b increases during MK differentiation and PLT production

**Figure 1:**
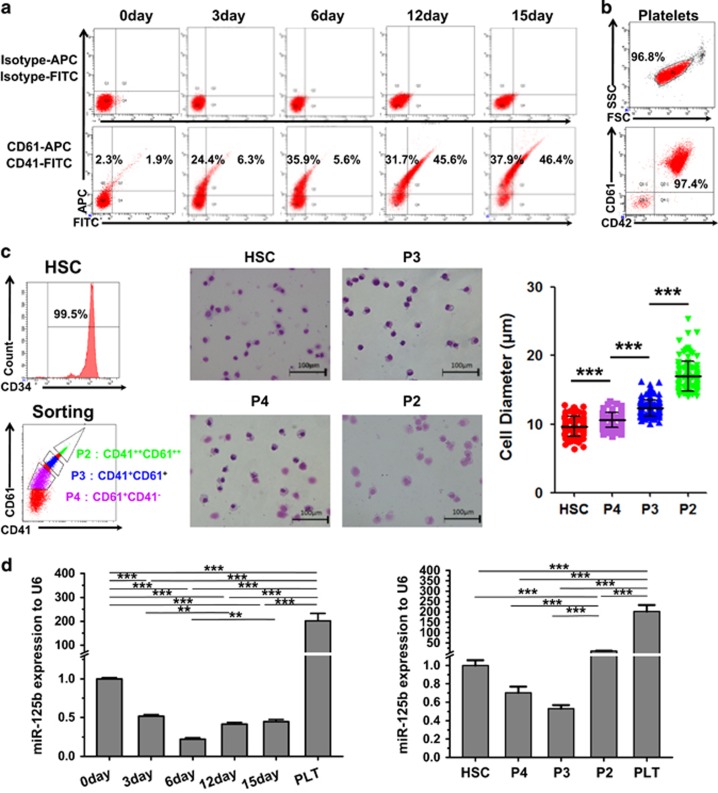
The upregulation of miR-125b is correlated with MK determination and maturation. (**a**) CD34^+^ hematopoietic cells were differentiated to MKs by culture in a megakaryocytic differentiation medium. The proportion of CD41^+^/CD61^+^ cells is indicated. (**b**) Isolated PLT from CB samples highly express the surface markers CD61 and CD42. (**c**) Isolation of CD34^+^ hematopoietic cells and megakaryoblasts at different stages of development was performed by cell sorting based on the expression levels of surface markers. Morphological difference between different stages was shown by Wright–Giemsa staining. Over 100 cells from five random views were measured. The error bars represent standard deviation (S.D.). Dunnett's test *T*3 were used for statistical analysis. (**d**) MiR-125b expression varies in different MK differentiation stages and during each megakaryocytic induction time. MK-specific induction starts with primary human HSCs. Changes in miR-125b were evaluated by qPCR. Comparative miRNA real-time PCR was performed in triplicate, and the expression levels were normalized to U6 miRNA. The error bars represent S.D. All of the data are expressed as the mean±S.D. from three experiments. Cells were obtained from three different donors. Bonferroni's multiple comparison test was used for statistical analysis. ***P<0.001 and **P<0.01

To gain insight into the role of miR-125b in MK differentiation, we investigated the endogenous expression of miR-125b in HSC-MK differentiation. First, we examined the total cellular expression of miR-125b in differentiating megakaryoid cells. RNA was collected every 3 days, starting from day 0 over 15 days of induction. To evaluate megakaryocytic maturation and enrichment, we analyzed the expression of the megakaryocytic surface markers CD41 (GPIIb) and CD61 (GPIIIa/*β*3). The elevated expression of these MK biomarkers (CD41 and CD61) in the cultures is shown in Figure 1a. miR-125b expression decreased during the first 6 days of *in vitro* differentiation, and then slightly increased after day 6 of culture. The expression of miR-125b was markedly elevated in PLTs isolated from cord blood (CB) (Figure 1b) (>200-fold) compared with undifferentiated CD34^+^ hematopoietic cells (Figure 1d, left panel). Although miR-125b expression in primary cells exhibited a certain degree of variability among the individual donors, it progressively and markedly increased in PLTs in all of the samples analyzed.

We then attempted to separate mature and immature MKs based on the different fluorescence intensity of the MK markers. CB cells cultured for 15 days were chosen for FACS sorting. Three populations were separated according to the expression levels of CD41 and CD61 (Figure 1c). These populations are designated as: CD41^hi^CD61^hi^ (CD41^++^CD61^++^, P2), CD41^med^CD61^med^ (CD41^+^CD61^+^, P3) and CD41^med^CD61^low^ (CD61^+^, P4). Sorted MKs at distinct stages of development, starting from CD34^+^ hematopoietic cells to cells cultured for 15 days, also present morphological differences. From HSC to P2, the cell size increased and the nucleus of the MKs became larger and lobulated. miR-125b expression levels were low in the early stages of differentiation, and progressively increased until the terminal phases of maturation. Compared with CD34^+^ HSCs, we also found an enrichment (10-fold) of miR-125b in CD41^hi^CD61^hi^ late-stage MKs, suggesting that miRNA-125b might be involved in the common process of MK terminal differentiation and PLT generation (Figure 1d, right panel).

**Figure 2:**
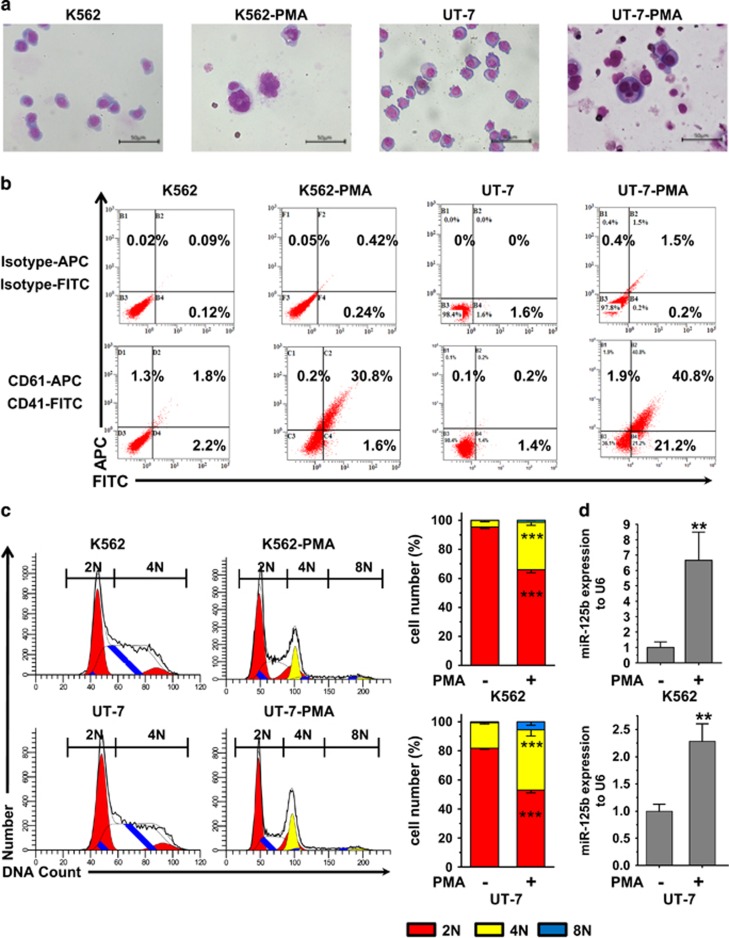
MiR-125b is enriched in PMA-induced K562 and UT-7 megakaryocytic cells. PMA treatment induces megakaryocytic differentiation of K562 and UT-7 cells. (**a**) Morphology of K562 and UT-7 cells before and after PMA treatment. Cells were stained with Wright–Giemsa solutions. (**b**) Flow cytometry analysis of CD41 and CD61. (**c**) DNA ploidy analysis by flow cytometry of K562 and UT-7 cells. In the ploidy mode, first cell cycle represents diploidy, which is shown by red color. Second cycle is polyploid, which color is yellow. White peaks and peaks filled with blue lines represent the S phase. Cells in the G1 and S phase of diploidy are 2N as judged by chromosome count. 4N is the sum of the G2 and M phase from diploidy and the G1 and S phase from polyploidy. 8N is the G2 and M phase of polyploidy. 2N-PMA *versus* 2N+PMA and 4N-PMA *versus* 4N+PMA show significant difference. DNA ploidy up to 8N only can be observed in PMA-treated cells (2N, red; 4N, yellow; 8N, blue). (**d**) qPCR analysis of miR-125b levels in undifferentiated or PMA-treated K562 and UT-7 cells. The endogenous expression of miR-125b in megakaryocytic cells increases after PMA treatment. U6 was used as an endogenous miRNA expression control. All of the data are expressed as the mean±S.D. from at least three independent experiments. Student *t*-tests were used for statistical analysis. ***P<0.001 and ** *P*<0.01

K562 and UT-7 cells can be induced to differentiate into cells with megakaryoblastic characteristics by phorbol myristate acetate (PMA) treatment.^7, 8^ K562-PMA and UT-7-PMA models were used to validate the results obtained from human CD34^+^ CB hematopoietic cells. We compared miR-125b expression in undifferentiated *versus* PMA-treated cells. After PMA treatment, K562 and UT-7 cells stopped proliferating, became larger and polyploid and expressed the MK markers CD41 and CD61 (Figures 2a–c). This phenotype suggests that PMA treatment facilitates the production of MKs. When comparing miRNA expression in untreated cells and in cells cultured for 3 days with PMA, an upregulation of miR-125b by 6.5-fold was observed after PMA treatment in K562 cells (Figure 2d, top panel). Similarly, megakaryocytic differentiation of UT-7 cells resulted in the enrichment (10-fold) of miR-125b (Figure 2d, bottom panel). Thus, MK differentiation of HSCs, K562 cells and UT-7 cells was associated with an increased expression of miR-125b. These observations suggest that miR-125b might be positively associated with megakaryocytic differentiation.

### Effect of miR-125b overexpression or downregulation on MK differentiation

**Figure 3:**
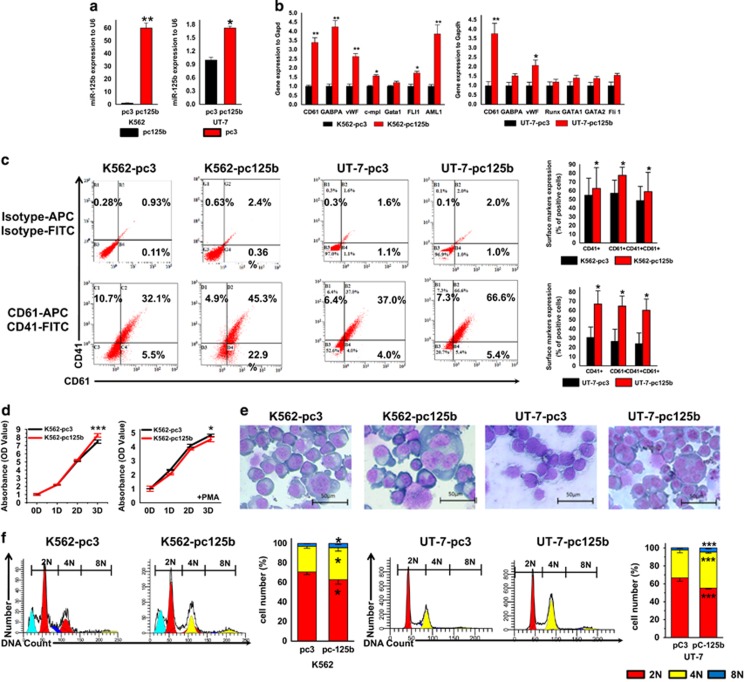
Overexpression of miR-125b promotes megakaryocytic differentiation and polyploidization of K562 and UT-7 cells. (**a**) In K562 and UT-7 cells, transfection with pcDNA3.1-pri-miR-125b effectively increases miR-125b expression compared with the pcDNA3.1 vector transfection control. (**b**) In K562- and UT-7-stable transfectants, overexpression of miR-125b upregulates the MK-specific genes when treated with PMA. Relative expression of MK marker genes was analyzed by qPCR. (**c**) The effect of the miR-125b expression construct on the percentage of MKs (CD41^+^CD61^+^). Representative flow cytometry plots are shown. (**d**) CCK8 assay was used to evaluate the proliferation of the miR-125b-modified K562 cells with (right panel) or without (left panel) PMA treatment. (**e**) In K562- and UT-7-stable transfectants, miR-125b overexpression facilitates the occurrence of megakaryocytic morphology after PMA treatment. Cytospin-prepared MKs were stained with Wright–Giemsa solutions. (**f**) Ploidy status of PMA-treated cells was assessed by flow cytometry after PI staining. A representative experiment is shown on the right panel, as well as the mean (±S.D.) percentage of cells in each ploidy (2N, red; 4N, yellow; 8N, blue). All of the data are expressed as the mean±S.D. from four independent experiments. *T*-tests were used for statistical analysis. *P<0.05, **P<0.01 and ***P<0.001 (scale bars: 50 *μ*m)

Endogenous expression of miR-125b is low in two human leukemia cell lines, K562 and UT-7. Therefore, we overexpressed miR-125b in K562 and UT-7 cells to determine whether the upregulation of miR-125b observed during MK differentiation has a role in controlling MK maturation. Successful overexpression of mature miR-125b in both cell lines is shown in Figure 3a. Subsequently, the cells were cultured in medium containing 1 nM PMA to induce megakaryotic differentiation and maturation. Overexpression of miR-125b promoted the expression of MK integrins (Figure 3c). Another indicator of MK differentiation is the expression of genes important for MK lineage, which regulate lineage-specific differentiation of MKs and characterize the maturation of human MKs (megakaryocytic genes). Similarly to what was observed by flow cytometry, higher expression of CD61 and other important megakaryocytic genes was also detected by quantitative RT-PCR (qPCR) in pcDNA3.1-pri-miR-125b cells when compared with control cells, which is consistent with previous observations (Figure 3b).

Cessation of cell proliferation is a hallmark of terminal differentiation.^9^ Hence, we examined whether cell proliferation was altered by miR-125b overexpression. At 72 h after PMA treatment, the cell amplification blockage resulting from miR-125b overexpression was more remarkable in K562 cells (Figure 3d). And after additional 6 days of treatment, the increase in cell size and ploidy level was evident (Figure 3e). Ploidy analysis by flow cytometry showed that although most of the polyploid cells resulting from PMA treatment were 4N, which indicates immature MK progenitors, the percentage of 4N cells increased in pcDNA3.1-pri-miR-125b/K562 cells (62.99%) and pcDNA3.1-pri-miR-125b/UT-7 cells (40.43%) *versus* pcDNA3.1/K562 cells (40.68%) and pcDNA3.1/UT-7 cells (31.12%), respectively (Figure 3f).

**Figure 4:**
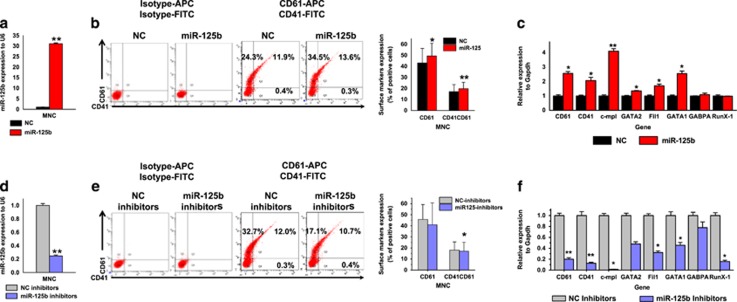
Effect of miR-125b mimics and inhibitors on MK differentiation of human umbilical CB MNCs. Cells were cultured in a megakaryocytic differentiation medium for 6 days, and transiently transfected with miR-125b mimics or inhibitors at day 1. (**a**) Relative miR-125b expression analyzed by qPCR. miR-125b expression is normalized to U6. (**b**) Overexpression of miR-125b in MNCs induces the expression of the MK integrins CD41 and CD61. (**c**) Relative expression of MK marker genes. Glyceraldehyde 3-phosphate dehydrogenase (GAPDH) was used as a housekeeping control in the experiment. qPCR results from cells transfected with NC mimics are assigned an arbitrary value of 1. (**d**) Transient transfection of the designated inhibitor effectively downregulates the expression of miR-125b. (**e**) Downregulated miR-125b expression in MNCs reduces the expression of the MK integrins CD41 and CD61. (**f**) Relative expression of MK marker genes. GAPDH was used as a housekeeping control in the experiment. qPCR results from cells transfected with NC inhibitor mimics are assigned an arbitrary value of 1. Cells were obtained from four different donors and each sample was tested with three independent experiments for data presentation. All of the data are expressed as mean±S.D. Student's *t*-tests were used for statistical analysis. *P<0.05 and **P<0.01

We further determined whether miR-125b also contributes to MK differentiation of primary human hematopoietic stem and progenitor cells. For this purpose, miR-125b expression was modified in human CB mononuclear cells (MNCs). The cells were grown in megakaryocytic differentiation medium and transfected on day 1 with miR-125b mimics or non-target negative control (NC) mimics. Transfection of miR-125b mimics increased miR-125b levels of is ~30-fold compared with NC cells (Figure 4a). Then, we evaluated the effect of miR-125b overexpression on MK markers and megakaryocytic genes. After 6 days of culture, miR-125b-transfected MNCs showed a higher CD61^+^ (49.4% *versus* 43.1%) and CD41^+^/CD61^+^ percentage (20.6% *versus* 17.9%) than NC cells (Figure 4b). The mRNA expression of MK glycoproteins (CD41 and CD61) increased >2-fold. Moreover, qPCR results showed that the expression of MK transcription factors (GABPA, GATA-1, GATA-2, FIL1 and RUNX) and thrombopoietin (TPO) receptor (C-MPL) increased accordingly (Figure 4c).

Subsequently, MNCs were transfected with miR-125b inhibitor mimics or non-target NC inhibitors. By using specific inhibitors, miR-125b expression was successfully downregulated over fivefold in MNCs (Figure 4d). As expected, the miR-125b inhibitors retarded MK differentiation. The percentage of CD61^+^ and CD41^+^/CD61^+^ populations in MNCs transiently transfected with miR-125b inhibitors was 36.3% and 14.5%, whereas in the NC inhibitor control cells, the corresponding percentages were 41% and 15.6%, respectively (Figure 4e). qPCR analysis revealed that treatment with miR-125b inhibitors repressed megakaryocytic genes when compared with the NC inhibitor group (Figure 4f). In summary, the overexpression of miR-125b increases MK differentiation of MNCs, whereas the suppression of miR-125b inhibited MK differentiation. However, the effect of miR-125b overexpression in MNCs was not as robust as in K562 or UT-7 cells.

### Alteration of miR-125b in human HSCs, CFU-MK cells and pro-MKs exerts different effects on MK differentiation

The positive effect of miR-125b on megakaryopoiesis has been elucidated previously.^4^ However, modification of miR-125b activity in MNCs did not present as a profound impact on MK differentiation as reported. The heterogeneity of MNCs might contribute to the unclear effect observed in this study. Because the endogenous miR-125b expression varies depending on the differentiation stage of MKs (Figure 1), a stage-specific effect of miR-125b was anticipated and analyzed.

**Figure 5:**
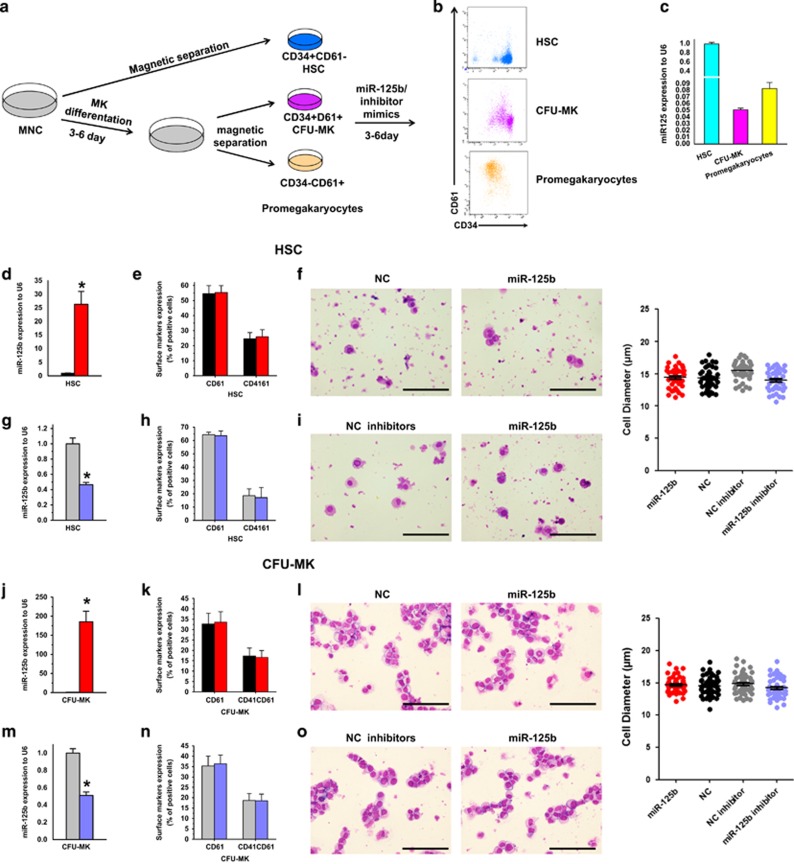

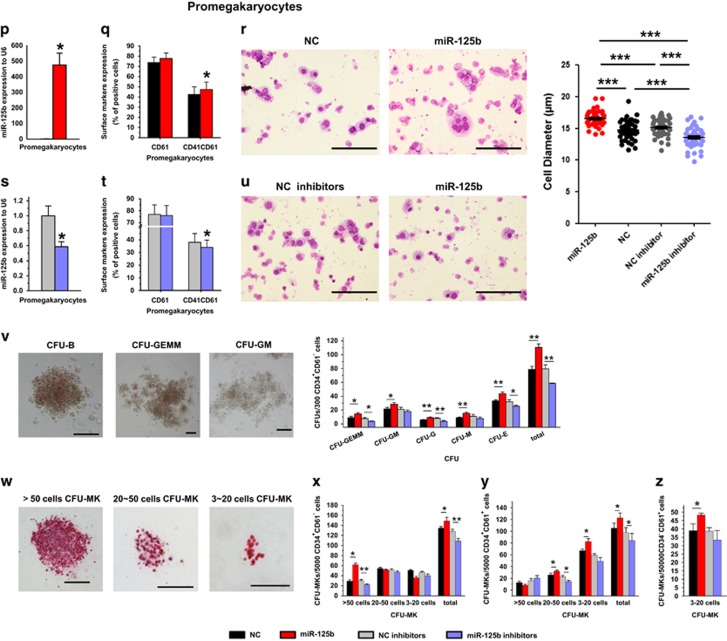
Alteration of miR-125b in human hematopoietic stem/progenitor cells, CFU-MK cells and pro-MKs have different effects on MK differentiation. (**a**) Schematic illustration of the experimental setup and the expression of differentiation markers during human megakaryocytic differentiation. (**b**) The expression of CD34 and CD61 on cells isolated by magnetic sorting, which represent different developmental stages of human MKs. (**c**) qPCR analysis of miR-125b expression at different stages of MK differentiation. (**d**, **g**, **j**, **m**, **p** and **s**) Relative miR-125b expression analyzed by qPCR. miR-125b expression is normalized to U6. (**e**, **h**, **k**, **n**, **q** and **t**) Altered expression of miR-125b influences the expression of MK integrins CD41 and CD61. (**f**, **i**, **l**, **o**, **r** and **u**) Altered expression of miR-125b impacts on megakaryocytic morphology and cell dimension. Cytospin-prepared MKs were stained with Wright–Giemsa solutions. Over 50 pro-MKs from five random views were measured. The error bars represent standard deviation (S.D.). Dunnett's test *T*3 were used for statistical analysis. (**v**) The typical morphology of CFU-E, CFU-GM, CFU-GEMM and quantification of CFUs with methylcellulose-based colony-forming assays of miR-125b, miR-125b inhibitor, NC and NC inhibitor-modified HSPCs. (**w**) Different size of CFU-MK colonies stained with human CD41 antibody. (**x**–**z**) Calculation of CFU-MKs with megacul colony-forming assays of miR-125b, miR-125b inhibitor, NC and NC inhibitors modification at different stages of human MK differentiation. Cells were obtained from four different donors and each sample was tested with three independent experiments for data presentation. All of the data are expressed as mean±S.D. Student's *t*-tests were used for statistical analysis. *P<0.05, **P<0.01 and ***P<0.001 (scale bars: 100 *μ*m)

During megakaryopoiesis, the expression of CD61 showed the most marked and progressive increase over time, while surface expression of CD34 decreased.^10^ Using well-established surface markers, we purified three populations of cells. We refer to them as: HSCs CD34^+^CD61^−^, neonatal MK progenitors (CFU-MKs) CD34^+^CD61^+^ and mature MK progenitors (pro-MKs) CD34^−^CD61^+^. Magnetic cell sorting (MACS) technology was used to isolate the three populations from human CB MNCs before or after MK differentiation *in vitro*. After separation, we stained the cells with antibodies against CD34 and CD61, and examined the endogenous expression of miR-125b (Figures 5a–c). Subsequently, miR-125b expression was modified in HSCs, CFU-MKs and pro-MKs (Figures 5d, g, j, m, p and s), followed by 5 days MK-specific induction and detection.

For HSCs and CFU-MKs, gain or loss of function of miR-125b did not result in a significant alteration of the percentage of CD61^+^ and CD41^+^ populations and cellular morphology after the induction of differentiation (Figures 5d–o and Supplementary Figures S1A and B).

Studies with promegakryocytes demonstrated that both early (CD41 and CD61) and late (CD42b) megakaryocytic surface markers could be detected by flow cytometry after induction. And the overexpression of miR-125b promoted the expression of all these MK differentiation markers (Figure 5q and Supplementary Figure S1C). When comparing MK maturity by cellular morphology, pro-MKs with miR-125b mimics show more mature MK phenotypes, the huger and higher polyploid (8N or more), than NC mimics (Figure 5r). Moreover, miR-125b inhibitor significantly retarded MK differentiation as assessed by comparing cellular morphology and expression of the megakaryocytic surface markers (Figures 5t–u). Concomitantly, we evaluated the number of PLT released in culture media. From the same number of seeded pro-MKs, we obtained more PLTs in miR-125b-transfected pro-MKs than the NC cells and less in miR-125b decreased expression than with NC inhibitor (Supplementary Figures S1F–G).

To confirm the function of miR-125b at distinct stages of MK development, a CFU-MK colony formation assay was also performed to quantitate the megakaryocytic potential of the progenitor cells. As we known, neonatal MK progenitors are hyperproliferative and generate more MKs per colony, although the colony cells are smaller and less polyploid compared with adult progenitors that generate more PLTs per MK.^11^ As a consequence, large MK colonies arise from more primitive MK progenitors, whereas smaller MK colonies are produced by more mature MK progenitors. Therefore, we subdivided the colonies based on their size: small (3–20 cells per colony), medium (21–49 cells per colony) and large (⩾50 cells per colony). We found that in HSCs, forced miR-125b expression resulted in more CFU-GEMM and large CFU-MK units, but had no effect on medium and small CFU-MK units (Figures 5v–x), suggesting a uniform effect of miR-125b on all the myeloid lineages at this stage. Sorted CFU-MKs and pro-MKs could not derive myeloid colonies except for megakaryocytic colonies (data not shown). For these cells, miR-125b overexpression resulted in a statistically significant increase in total MK colony-forming units, especially in small CFU-MK units, but showed no effect on large CFU-MK units. In contrast, loss of miR-125b function showed a marked decrease in megakaryocytic colony formation (Figures 5w–z). In conclusion, these gain- and loss-of-function experiments strongly suggest that miR-125b promotes MK differentiation at a late stage of maturation.

### The G1 cell-cycle inhibitor p19^INK4D^ is a target of miR-125b during megakaryopoiesis

**Figure 6:**
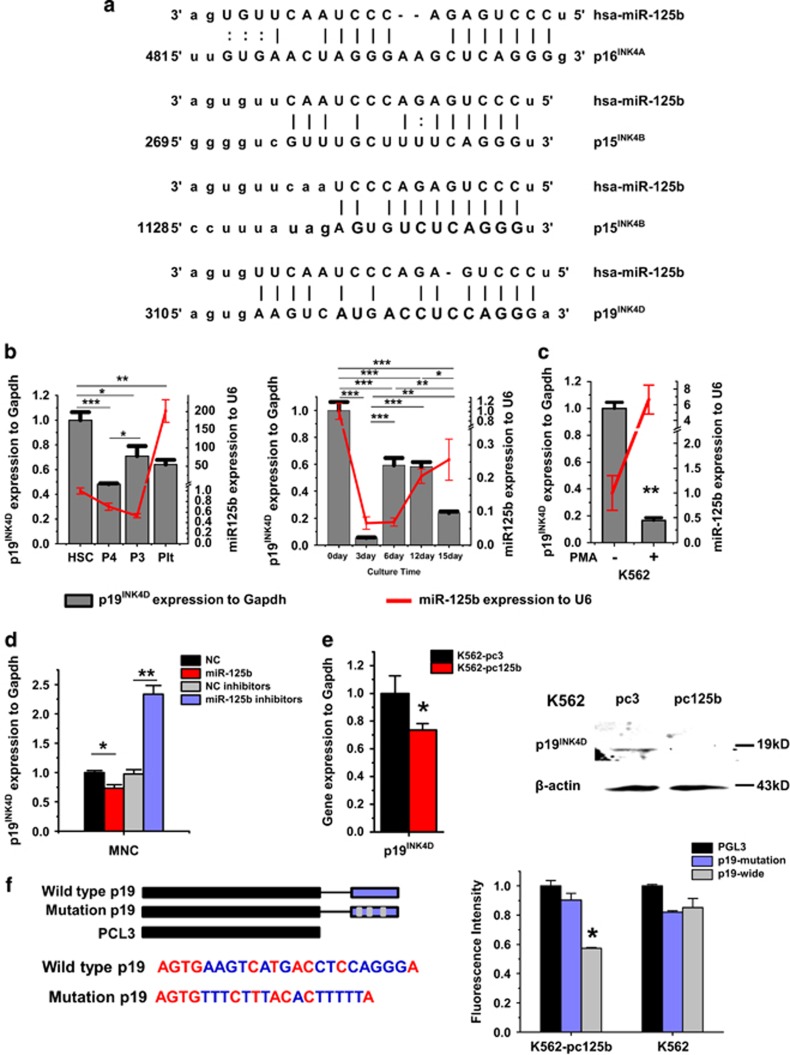
Scan for miR-125b target genes during MK differentiation. P19^INK4D^ is a direct target of miR-125b. (**a**) Bioinformatic analysis of the potential interactions between miR-125b and the 3′-UTR of genes from the INK4 family. (**b**) The expression pattern of p19^INK4D^ during MK differentiation, as well as at distinct stages of maturation. The megakaryocytic induction begins with CD34^+^ hematopoietic precursors. (**c**) qPCR analysis of p19^INK4D^ levels in untreated or PMA-treated K562 cells. Glyceraldehyde 3-phosphate dehydrogenase (GAPDH) was used as an endogenous expression control. (**d**) P19^INK4D^ is upregulated upon miR-125b silencing; in contrast, p19^INK4D^ is efficiently downregulated after miR-125b overexpression. (**e**) P19^INK4D^ expression at the mRNA and protein levels in K562 cells stably transfected with pcDNA3.1 (control) or pcDNA3.1-pri-miR-125b were evaluated by qPCR and western blot, respectively. (**f**) Putative miR-125b binding sequence in the p19^INK4D^ 3′-UTR, and the designed scheme for wild-type and mutant recognition fragments from p19^INK4D^ 3′-UTR. Relative luciferase activity indicates direct binding and function of miR-125b on the p19^INK4D^ 3′-UTR. miR-125b downregulates p19^INK4D^ by interacting with its 3′-UTR. Relative repression of firefly luciferase activity was normalized to a transfection control. Individual comparisons between each groups were performed using Student's paired *t*-test. Bonferroni's multiple comparison test for multiple comparisons was applied. *P<0.05, **P<0.01 and ***P<0.001

*In silico* prediction of miRNA targets suggests that thousands of mRNAs could be regulated by miR-125b at the transcriptional or post-transcriptional level. Few studies have noted that miR-125b has different functions in different cellular contexts.^12^ In megakaryocytopoiesis, a previous report^5^ and our study both demonstrated the important role that miR-125b has in MK endomitosis, which results in the polyploidy of MKs. Accordingly, we narrowed our analysis of potential miR-125b targets in megakaryopoiesis to cell-cycle regulators, especially to the INK4 family, whose members have been suggested to have a pivotal role in G1 cell-cycle suppression and MK endomitosis.^13^ By consulting relevant literatures and performing integrative bioinformatic analysis, we identified potential miR-125b binding sites in the 3′-UTRs of p16^INK4A^, p15^INK4B^ and p19^INK4D^ (Figure 6a). We had demonstrated the upregulation of miR-125b was required for the maturation of MKs, as a consequence, its putative targets should be downregulated during this process. During human MK differentiation, an upregulation of p15^INK4B^ ^14^ and a downregulation of p16^INK4A^ have been observed.^15, 16^ Thus, we only analyzed the endogenous expression of p19^INK4D^ in CD34^+^ hematopoietic cells during MK differentiation and PLT production. We found that the expression of p19^INK4D^ first increased and then decreased. In particular, p19^INK4D^ mRNA also decreased in PLTs and in CD41^hi^CD61^hi^ late-stage MKs (Figure 6b). Similarly, following PMA treatment, mRNA expression of p19^INK4D^ decreased during MK differentiation (Figure 6c). Further, a negative correlation between the expression of miR-125b and p19^INK4D^ was shown. Next, we sought to verify the regulation of p19^INK4D^ by miR-125b. Overexpressing miR-125b in CD34^+^ hematopoietic cells decreased the mRNA expression levels of p19^INK4D^. A remarkable increase of p19^INK4D^ mRNA was observed in cells transfected with miR-125b inhibitors compared with cells transfected with NC inhibitors (Figure 6d). Protein and mRNA expression levels of p19^INK4D^ in pcDNA3.1-pri-miR-125b/K562 cells were considerably lower compared with that in pcDNA3.1 cells (Figure 6e). Therefore, p19^INK4D^ is one of the putative target genes of miR-125b, which has an identical seed sequence complementary to the binding site on the 3′-UTR of p19^INK4D^. Using a luciferase reporter assay, we demonstrated that p19^INK4D^ is a direct target of miR-125b. miR-125b overexpression significantly decreased the luciferase activity of the reporter containing the wild-type 3′-UTR of p19^INK4D^, when compared with the mutant form and the controls (Figure 6f).

### Overexpression or knockdown of p19^INK4D^ inhibits or promotes MK differentiation, respectively

**Figure 7:**
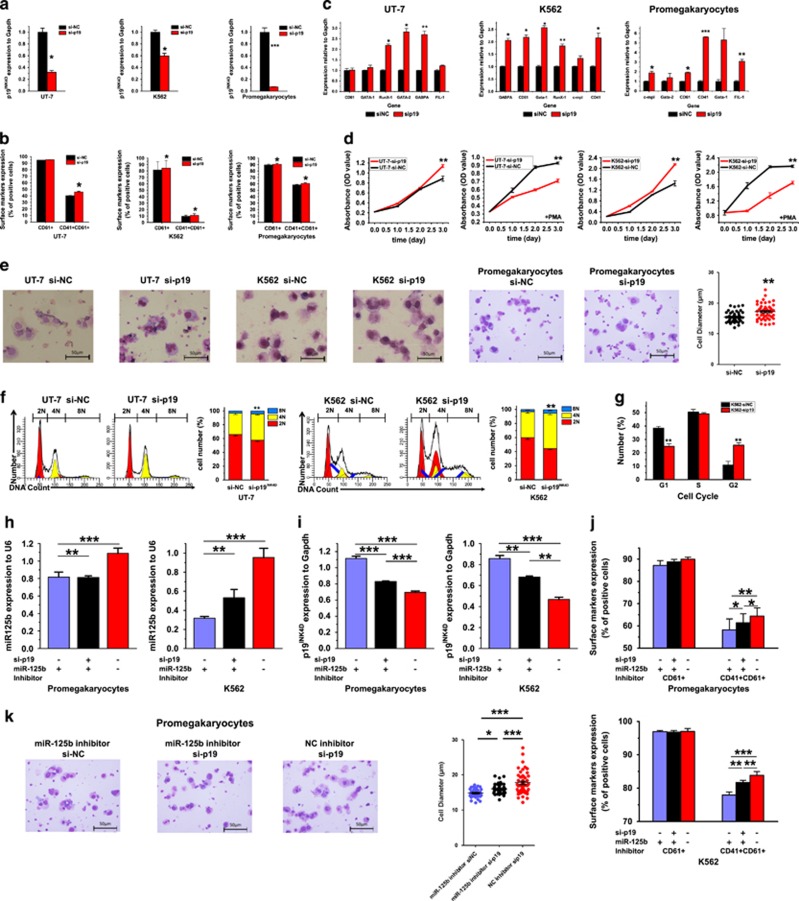
Direct downregulation of p19^INK4D^ promotes MK differentiation of K562 cells, UT-7 cells and pro-MKs. (**a**) Cells are transiently transfected with the indicated siRNA mimics for 24 h. Relative p19^INK4D^ expression was analyzed by qPCR. (**b**) K562 and UT-7 cells were transfected with the indicated siRNA and then treated with a suboptimal amount of PMA (1 nM) for 3 days. Megakaryocytic marker genes were analyzed by qPCR. (**c**) Differentiation markers were analyzed by flow cytometry. (**d**) Cell proliferation capacity of K562 and UT-7 cells transfected with sip19^INK4D^ or a control siRNA mimics (in the presence or absence of PMA treatment) was measured by CCK8 assay. (**e**) MK morphology. Cytospin-prepared MKs were stained with Wright–Giemsa solutions. Over 50 pro-MKs from five random views were measured. The error bars represent standard deviation (S.D.). (**f**) Ploidy status of PMA-treated K562 cells was assessed by flow cytometry after PI staining. A representative experiment is shown on the right panel, as well as the mean (±S.D.) percentage of cells in each ploidy (2N, red; 4N, yellow; 8N, blue). (**g**) Percentage of K562 cells in each cell-cycle phase. (**h** and **i**) Histograms of miR-125b and p19^INK4D^ mRNA in K562 cells or pro-MKs 48 h after co- transfection with miR-125b inhibitor mimics/NC inhibitors and p19^INK4D^ siRNA/control siRNA. (**j**) Differentiation markers were analyzed by flow cytometry. (**k**) MK morphology. Cytospin-prepared MKs were stained with Wright–Giemsa solutions. Over 50 pro-MKs from five random views were measured. The error bars represent standard deviation (S.D.). Dunnett's test *T*3 were used for statistical analysis. Cells were obtained from four different donors and each sample was tested with three independent experiments for data presentation. The results are presented as mean±S.D. Individual comparisons between two groups were performed using Student's paired *t*-test. Bonferroni's multiple comparison test for multiple comparisons was applied. *P<0.05, **P<0.01 and ***P<0.001 (scale bars: 50 *μ*m)

P19^INK4D^ is an important regulator of cell-cycle progression. Previous reports showed that p19^INK4D^ expression is correlated with endomitotic arrest and MK maturation and regulated by AML-1.^17^ Taken together with the results that p19^INK4D^ mRNA level decreased not only during MK differentiation but also in PLTs, we speculated that this molecule is involved in the arrest of MK terminal differentiation. To confirm our hypothesis, two human leukemia cell lines, K562 and UT-7 cells and CB-derived CD61^+^ pro-MKs were transfected with siRNAs against p19^INK4D^, and examined the effect of RNAi-mediated knockdown of p19^INK4D^ (Figure 7a). After siRNA transfection and megakaryocytic induction, sip19^INK4D^-transfected cells showed higher CD41^+^/CD61^+^ rate compared with control cells (Figure 7b and Supplementary Figures S2A and B). Moreover, the transcriptional levels of megakaryocytic genes also increased upon p19^INK4D^ knockdown (Figure 7c).

Cell-cycle variation is a key feature of endomitosis. We next analyzed cell proliferation and polyploid level after p19^INK4D^ knockdown. In agreement with the effect observed following miR-125b overexpression, cell proliferation was significantly reduced and the cell size and ploidy level was increased along with the decreased expression of p19^INK4D^ during MK differentiation (Figures 7d and e). Furthermore, ploidy analysis showed that p19^INK4D^ reduction increased the percentage of 4N and 8N cells (Figure 7f) together with the G1-phase abatement (Figure 7g).

We next examined whether miR-125b inhibition and subsequently p19^INK4D^ upregulation as well as MK differentiation blockage could be antagonized by direct p19^INK4D^ knocking down (Figure 7h). Both in K562 cells and pro-MKs, p19^INK4D^ siRNA treatment partially rescued the elevation of p19^INK4D^ mRNA, resulting from miR-125b inhibitor transfection (Figure 7i). The antidifferentiation effects of miR-125b inhibitor in megakaryocytic surface markers and cellular morphology were, in part, abrogated by the concomitant p19^INK4D^ downmodulation (Figures 7j and k and Supplementary Figures S2C and D).

**Figure 8:**
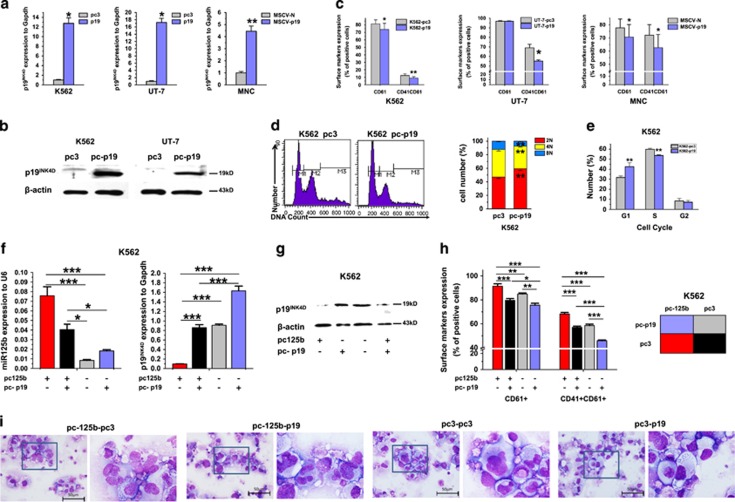
Enforced p19^INK4D^ expression reduces MK differentiation. (**a**) P19^INK4D^ expression was measured in K562 and UT-7 cells stably transfected with pcDNA3.1 (control) or pcDNA3.1-p19^INK4D^ and MNCs infected with HA-tagged p19^IK4D^-expressing retrovirus by qPCR. Error bars represent the standard deviation of the mean of three repeated experiments. (**b**) Western blot analysis of p19^INK4D^ expression in K562 and UT-7 cells overexpressing p19^INK4D^. (**c**) Overexpression of p19^INK4D^ reduced expression of MK markers in K562 cells, UT-7 cells and mononuclears, as assessed by flow cytometry. (**d**) DNA ploidy analysis by flow cytometry of K562 cells stably transfected with pcDNA3.1 (control) or pcDNA3.1-p19^INK4D^. A representative experiment is shown on the right panel, as well as the mean (±S.D.) percentage of cells in each ploidy (2N, red; 4N, yellow; 8N, blue). (**e**) Percentage of cells in each cell-cycle phase from p19^INK4D^-overexpressing K562 cells. (**f**) P19^INK4D^ and miR-125b expression were measured in K562 cells stably co-transfected with pcDNA3.1-puromycin/pcDNA3.1-p19^INK4D^-puromycin and pcDNA3.1-neomycin/pcDNA3.1-pri-miR-125b-neomycin by qPCR. (**g**) Western blot analysis of p19^INK4D^- and miR-125b-comodified K562 cells. (**h**) Flow cytometry analyses of MK marker expression. (**i**) MK morphology change after p19^INK4D^ and miR-125b comodification. All of the data are expressed as mean±S.D. from at least three independent experiments. Individual comparisons between two groups were performed using Student's paired *t*-test. Bonferroni's multiple comparison test for multiple comparisons was applied. *P<0.05 and **P<0.01 (scale bars: 50 *μ*m)

To confirm the role of p19^INK4D^ in MK differentiation and polypoidization, the effect of p19^INK4D^ upregulation on MK development was detected. For p19^INK4D^ overexpression, the expression plasmid pcDNA3.1-p19^INK4D^-puromycin was constructed and transfected into K562 and UT-7 cells. MNCs were infected with HA-tagged p19^IK4D^-expressing retrovirus. The upregulation of p19^INK4D^ was confirmed at both the mRNA and protein levels (Figures 8a, b and Supplementary Figure S3A). P19^INK4D^ overexpression significantly delayed MK differentiation, as assessed by the CD41^+^/CD61^+^ and MK polyploidization rate. Especially, 62.5% of the p19^INK4D^-transfected MNCs (HA^+^) were CD41/61^+^ on average *versus* 72.1% for the control virus-infected cells, which implies that p19^INK4D^ overexpression is capable of inhibiting MK differentiation (Figures 8c, d and Supplementary Figure S3C). The cell-cycle assay demonstrated that p19^INK4D^ inhibits cellular proliferation by increasing the G1 compartment (Figure 8e). When p19^INK4D^ expression was rescued by pcDNA3.1-p19^INK4D^ transfection in miR-125b-overexpressing K562 cells (Figures 8f and g), the MK promoting effects of miR-125b were, in part, abrogated by the complementary p19^INK4D^ overexpression (Figures 8h, i and Supplementary Figure S3D). Thus, we showed by integrative analysis that miR-125b may exert its effect on MK differentiation by repressing p19^INK4D^ expression.

## Discussion

MiR-125b is currently considered as a key molecule in the physiology and pathology of stem cell self-renewal and tumorigenesis. Ooi *et al.*^18^ and O'Connell *et al.*^19^ elucidated that miR-125b is a critical regulator of HSC self-renewal. It was also demonstrated by Klusmann *et al.*^4^ that miR-125b promotes MK self-renewal. Because miR-125b has been suggested to participate in both HSC self-renewal and MK differentiation, we tried to clarify the stage-specific effect of this molecule. Enhanced miR-125b expression promotes HSC self-renewal, as suggested by increased CFU content. Differentiation toward the myeloid lineage and MKs requires the downregulation of miR-125b over a specific period of time (Figure 1c). However, as soon as the determination of MKs has been achieved, miR-125b downregulation stops, and this miRNA keeps on accumulating until the final stages of MK differentiation, when PLTs are produced. It is reasonable to speculate that the intracellular microenvironment has an important role in the distinct function of miR-125b. In our study, the phase-specific function of miR-125b was clarified by stage-specific miRNA gene modification. Based on our findings, the specificity of miR-125b function to megakaryopoiesis should start at the late stages of MK development, in which miR-125b synergizes with MK-specific regulators (transcriptional factors, phosphorylated signal molecules and on on) to further accelerate the maturation of MKs. These cooperating factors remain to be determined in further studies.

Cell-cycle regulators have been reported to have an important role in hematopoiesis.^20^ We demonstrated that the levels of miR-125b and p19^INK4D^ varied synchronously during MK differentiation and maturation. miR-125b accumulates during the MK maturation phase, suggesting the pivotal role of this miRNA in MK development. The direct regulation of p19^INK4D^ by miR-125b during megakaryopoiesis was further confirmed by RT-PCR, western blot, luciferase reporter assay and target gene rescue experiments, implicating the suppression of p19^INK4D^ by miR-125b function in MK maturation. Although other INK4 family molecules, such as p15^INK4B^ and p16^INK4A^, have been confirmed to be crucial for the maturation of MKs,^21, 22, 23^ limited information is available of the role of p19^INK4D^ in MK maturation. A single study by Gilles *et al.*^24^ suggests that the increase in p19^INK4D^ expression is required for the final stages of MK maturation. However, this report also indicates that p19^INK4D^ levels were downregulated during 2N to 4N maturation, and MKs from p19^INK4D^ KO mice exhibited significant increased mean ploidy level. Considering the indispensable role of S-phase progression in MK polyploidization, as well as the effect exerted by p19^INK4D^ on arresting G1/S transition,^25, 26, 27^ it is possible that the polyploidization of MKs requires the downregulation of p19^INK4D^ to trigger the process. The key role of p19^INK4D^ for polyploidization initiation was confirmed by our study. When blocking the expression of p19^INK4D^ with shRNA, polyploidization accelerated, whereas p19^INK4D^ overexpression resulted in megakaryocytic differentiation blockage, as well as G1-phase arrest. As a conclusion, miR-125b improves the maturation and polyploidization of MKs by inhibiting p19^INK4D^ expression. However, the function of the accumulation of miR-125b in PLT remains to be determined. It is possible that the suppression of cyclin-dependent kinases by miR-125b mediates the final process of MK maturation.

Taken together, our findings contribute to elucidate the specific effect of miR-125b in MK development. miR-125b acts as a positive regulator of megakaryopoiesis, and supports the MK fate during all of the phase of HSC, MEP and MK differentiation. The effect on MK differentiation and polyploidization is, at least partially, mediated by the downregulation of p19^INK4D^. The application of miR-125b or small molecules that regulate its downstream signaling pathway might partially solve the shortage of PLT generation *ex vivo*, and meet the urgent requirement of PLT in the clinic. This study may contribute to the development of therapeutic strategies for PLT diseases.

## Materials and Methods

### Purification of human CB-derived CD34^+^ cells

Human CB samples were obtained from umbilical cord after the delivery of normal pregnancies with patient's informed consent. Research has been carried out in accordance with the approval of the ethics committee. CB samples were processed within 4 h. MNCs were isolated by Ficoll-Hypaque density gradient centrifugation (1.077 g/l; TBDscience, Tianjin, China). Then, CB-CD34^+^ cells were purified from the MNCs by magnetic force following the manufacturer's instructions (MACS; MiltenyiBiotec, Bergisch Glabach, Germany).

### Cell culture and reagents

Human erythroleukemia K562 cells were cultured in RPMI-1640 medium supplemented with 10% fetal calf serum. Human megakaryoleukemia UT-7 cells were cultured in RPMI-1640 medium supplemented with 10% fetal calf serum and 1 U/ml Epo. For MK differentiation, cells were treated with 1 nM PMA (Sigma) for 3 days.

Human umbilical CB MNCs and CD34^+^ HSCs were grown in megakaryocytic differentiation medium. The cells were cultured in StemSpan Defined Medium in the presence of a cytokine cocktail containing 50 ng/ml recombinant human (rh) TPO, 50 ng/ml rh stem cell factor, 20 ng/ml rh interleukin-3 (rhIL-3), 50 ng/ml rhIL-6 and 20 ng/ml rhIL-11.

For microscopy imaging, cells were spun on microscopic slides and stained with Wright–Giemsa solutions. NIKON microscope and NIS-Elements F software (Nikon Corporation,Tokyo, Japan) were used to capture picture and measure cell diameter.

### Cell proliferation assay

Cell proliferation was analyzed with Cell Counting Kit-8 (Dojindo Laboratories, Kumamoto, Japan). As described in the manufacturer's instructions, 100 *μ*l of K562 or UT-7 cell suspension (2 × 10^4^ cells per well) was dispensed in a 96-well plate. The cells were transfected by siRNA 1 day before the assay initiation. Subsequently, the plates were incubated with or without PMA (1 *μ*M) at different times (0, 24, 48 and 72 h). Ten microliters of the CCK8 solution was added to the well of the plate. After 2 h incubation at 37 °C, the absorbance at 450 nm was measured by a microplate reader.

### Antibodies

The following antibodies were used for either cell sorting or flow cytometry analysis: directly conjugated R-phycoerythrin (PE) anti-CD34 (eBioscience, San Diego, CA, USA); allophycocyanin (APC) anti-CD38 (eBioscience); fluorescein isothiocyanate (FITC) anti-CD41a (eBioscience); R-PE anti-CD41a (eBioscience); R-PE anti-CD42 (eBioscience); FITC anti-human CD61 (eBioscience); APC anti-CD61 (eBioscience). FITC-, APC- and R-PE-conjugated immunoglobulin G1 mAb (obtained from eBioscience) were used as isotype controls.

Western blots were probed with anti-p19^INK4D^, anti-*β*-actin or anti-HA antibodies purchased from Santa Cruz (Santa Cruz, CA, USA). *β*-Actin was used as a loading control.

### Flow cytometric analysis for cell cycle and polyploidy

Cells were collected and washed in PBS, followed by permeabilization with cold 70% methanol for preserving at −20 °C. Propidium iodide (50 *μ*g/ml) with RNAse were added to stain DNA in the cells for 30 min, and analyzed by BD FACS Calibur. Ploidy mode was used in polyploid analysis

### miRNA mimics and inhibitors transfection

Cells were seeded in 6-well plates and transfected by nucleofection with 4 *μ*g per well plasmid DNA, 1.6 *μ*g per well miRNA mimics (Genepharma, Shanghai, China), or non-target NC mimics according to the manufacturer's protocol.

### Quantitative RT-PCR

As described in the manufacturer's instructions, total RNA was isolated with TRIzol (Invitrogen, Carlsbad, CA, USA). Then, RNA (0.8 *μ*g) was reverse transcribed using a PrimeScript RT-PCR Kit (Takara Bio, Shiga, Japan). Quantitative RT-PCR was performed in triplicate using the SYBR Green Master Mix (Toyobo, Osaka, Japan) in a Real-Time PCR Machine (Bio-Rad, Hercules, CA, USA). All gene expression levels were normalized to the housekeeping gene *GAPDH*. Analysis of mature miRNA expression relative to U6 snRNA was performed using All-in-One qPCR Mix (GeneCopoeia, Rockville, MD, USA).The specific primers are listed in Supplementary Table 1.

### Dual-luciferase reporter assay

The cloned sequences and putative binding sites of miR-125b on p19^INK4D^ 3′-UTR are described in Figure 6. The sequences were cloned into the pGL3 control vector (Promega, Madison, WI, USA). For the luciferase reporter assay, stable miR-125b overexpressing or not K562 cells were transfected by 0.8 *μ*g plasmid (PGL3, wild-type p19^INK4D^-PGL3 or mutant p19^INK4D^-PGL3), together with the pRL *Renilla* luciferase vector (Promega) as an internal control. Luciferase activities were assayed 48 h post-transfection according to the manufacturer's protocol.

### Establishment of miR-125b and p19^INK4D^ overexpression cells

A 318-bp human pri-miR-125b2 coding sequence was amplified by polymerase chain reaction (PCR) from human MNC cDNA, and subcloned into a plasmid pcDNA3.1-neomycin. Stable miR-125b-overexpressing leukemia lines were developed using the recombinant plasmid (pcDNA3.1-pri-miR-125b2-neomycin). The empty plasmid (pcDNA3.1-neomycin), carrying a selection marker, the neomycin resistance gene, was used as a transfection control.

A 502-bp human p19^INK4D^ coding sequence (NM_001800.3) was amplified by PCR from human MNC cDNA, and subcloned into a plasmid pcDNA3.1-puromycin and retroviral vector MSCV-N-FLAG-HA-puromycin (Addgene, Cambridge, MA, USA).

### Retrovirus production, concentration and cell infection

Retrovirus was produced with plat-A cells, which are stably expressing virus packing proteins. The day before transfection, 5 × 10^6^ plat-A cells were plated in poly-l-lysine-coated 10 cm dishes. Twenty micrograms of retroviral vectors, MSCV-N-FLAG-HA-puromycin (MSCV-N) or MSCV-N-FLAG-HA-p19^INK4D^-puromycin (MSCV-p19), were used for transfection. Retroviral supernatant was concentrated by ultracentrifugation (26 000 r.p.m., 90 min). Titers of retrovirus were tested on 293T cells and HA and p19^INK4D^ expression was analyzed by western blot 3 days after infection (Supplementary Figure S3C). A total of 3 × 10^6^ MNCs were infected with high titer p19^IK4D^-expressing retrovirus overnight in 1 ml megakaryocytic differentiation medium with 8 *μ*g/ml polybrene. The medium was changed one day after transduction. HA and cellular surface marker was analyzed by flow cytometric analysis 15 days after transduction.

### Statistics

Selective statistic analysis is indicated in each experiment.

## Abbreviations

CFU-GEMM: colony-forming unit-granulocyte, erythrocyte, monocyte and megakaryocyte
HSC: hematopoietic stem cell
MACS: magnetic cell sorting
miRNA: microRNA
MK: megakaryocyte
NC: negative control
PCR: polymerase chain reaction
PLT: platelet
PMA: phorbol myristate acetate
QPCR: quantitative polymerase chain reaction
siRNA: small interfering RNA
UTR: untranslated region
