# Supplementary material for: miR-125b modulates megakaryocyte maturation by targeting the cell-cycle inhibitor p19INK4D
Source: Cell Death Dis. 2016 Oct 20;7(10):e2430–. doi: 10.1038/cddis.2016.288 (PMC5133966; doi:10.1038/cddis.2016.288)
Supplement: Supplementary Information [file cddis2016288x1.docx]

**Supplementary Information**

The supplementary information includes a table listing primers used in the study. Three figures primarily containing representative flow cytometric plots are contained as well.

Supplemental Table 1.

| Gene | Primer(5’-3’) |
| --- | --- |
| vWF | F： CCGATGCAGCCTTTTCGGA  R： TCCCCAAGATACACGGAGAGG |
| GATA-1 | F： TTGTCAGTAAACGGGCAGGTA  R：CTTGCGGTTTCGAGTCTGAAT |
| CD41 | F： GATGAGACCCGAAATGTAGGC  R： GTCTTTTCTAGGACGTTCCAGTG |
| CD61 | F： GTGACCTGAAGGAGAATCTGC  R： CCGGAGTGCAATCCTCTGG |
| c-mpl | F： CCAGCCAGGGGAACTTC  R： GCTTTGGTCCATCTTGCC |
| GATA-2 | F： CCCACCTTTTCGGCTTCCC  R： CATCTTCATGCTCTCCGTCAG |
| RUNXⅠ | F： TGCAAGATTTAATGACCTCAGGTTT  R： TGAAGACAGTGATGGTCAGAGTGA |
| p18^INK4C^ | F： AAACTTGGAAATCCCGAGATTGC  R： CGAAACCAGTTCGGTCTTTCAA |
| p19^INK4D^ | F： CTGCAGGTCATGATGTTTGG  R： CAGCAGTGTGACCCTCTTGA |


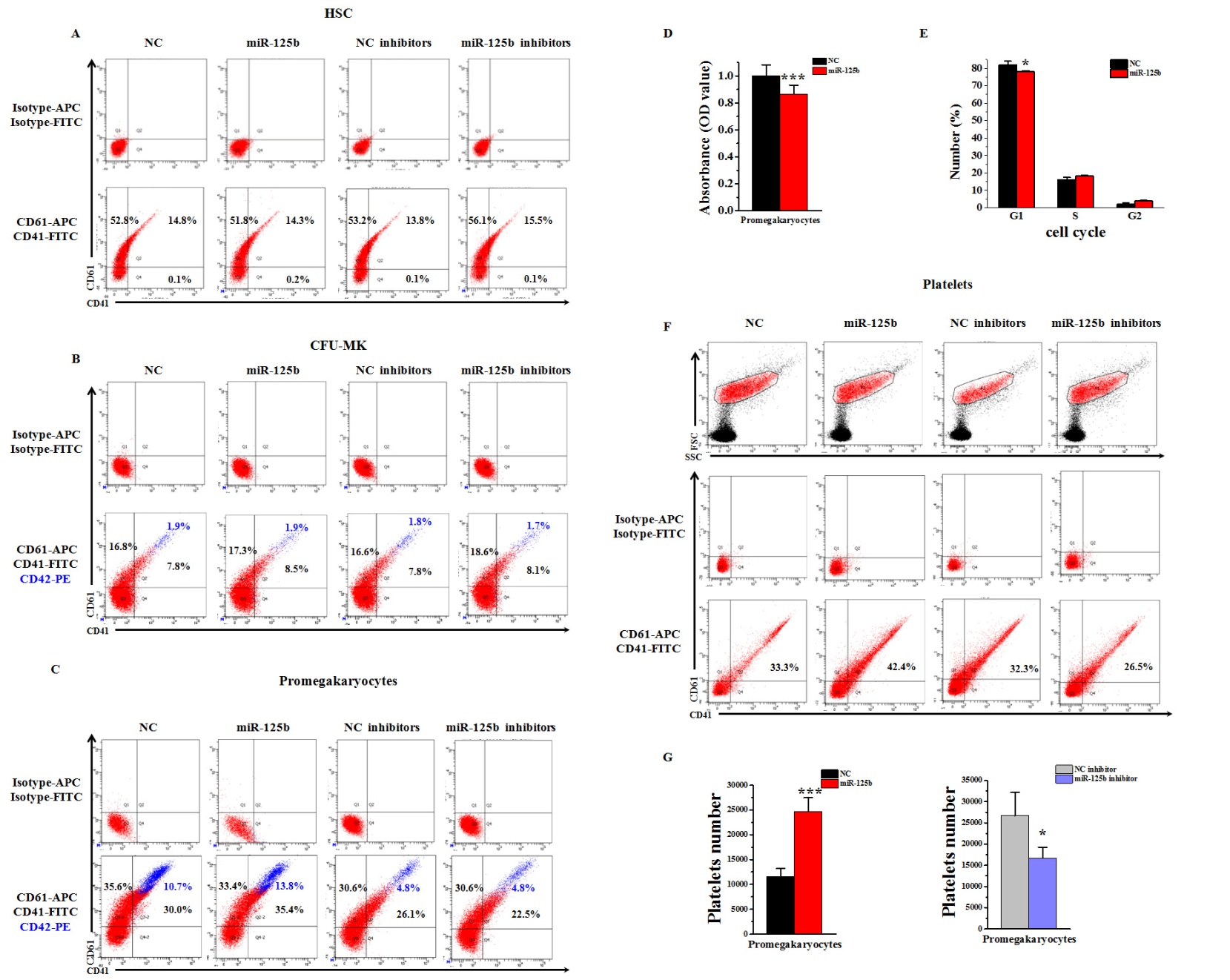
Figure S1. Stage-specific miR-125b modification exerts different impact on megakaryocytic surface marker expression, cell proliferation and platelet release.

(A-C) Flow cytometricanalysis of megakaryocyte differentiation from HSCs, CFU-MKs and promegakaryocytes respectively. The percentage of CD61+/CD41+ isshown. Blue dots represent CD41+/CD61+/CD42+ cells. (D) Cell proliferation of Promegakaryocytes transfected with miR-125b mimics or a negative control mimicswas measured by CCK8 assay.(E) Percentage of cultured promegakaryocytes in each cell cycle phase. (F)Dot plots demonstrate the gating strategyof flow cytometric platelet analyses.(G)Number of platelets released in culture media by the same number of promegakaryocytes. (D-G) are data harvested from promegakaryocytes with gene modification and megakaryocytic culture. Cells were obtained from 4 different donors and each sample was tested with 3 independent experiments for data presentation. All of the data are expressed as mean ± SD. Student t-tests were used for statistical analysis;* p<0.05; *** p<0.001.


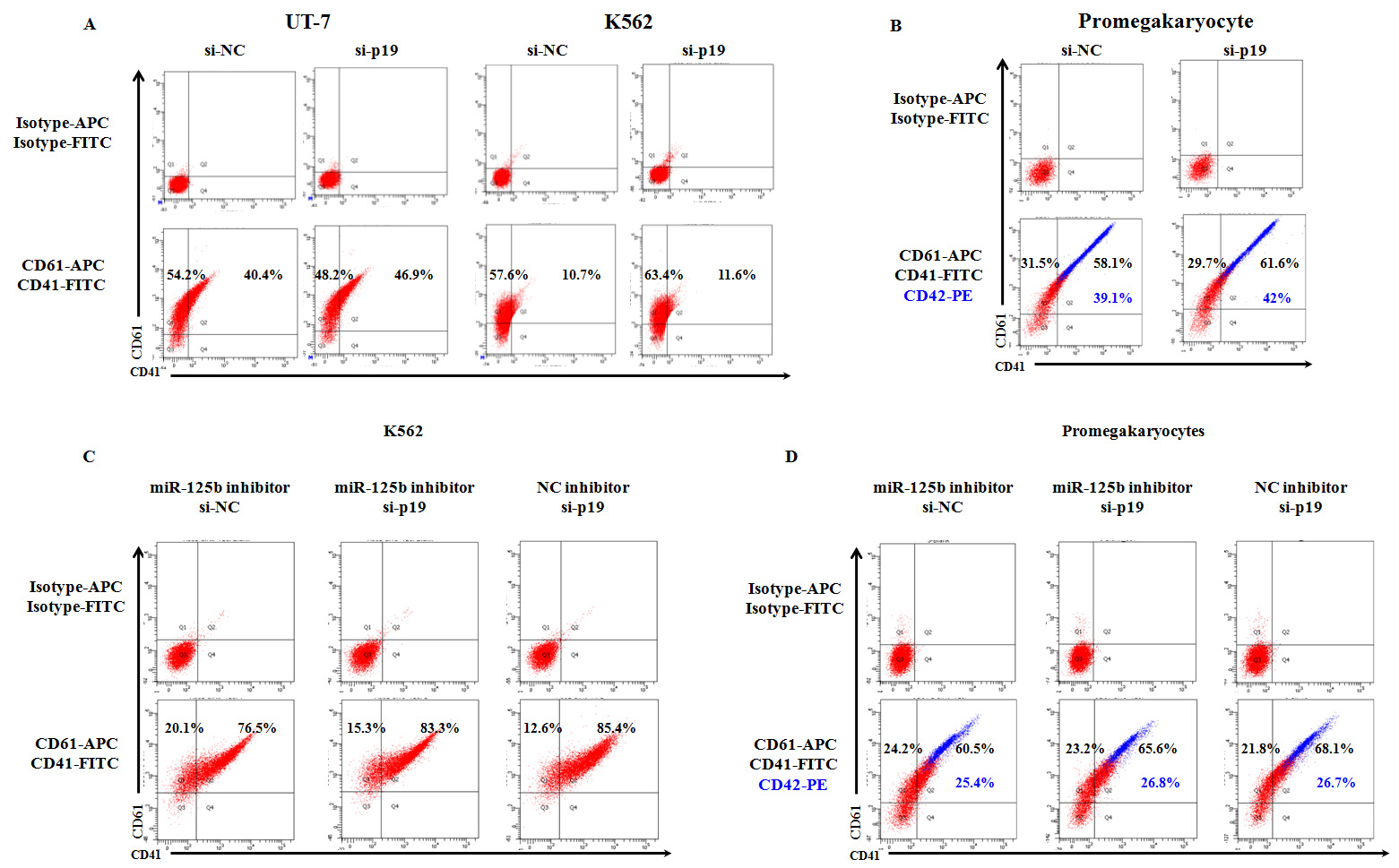
Figure S2. Representative flow cytometric plots demonstrate effects of p19^INK4D^ knock-down on megakaryocytic surface marker expression. The percentage of CD61+/CD41+ isshown. Blue dots represent CD41+/CD61+/CD42+ cells.


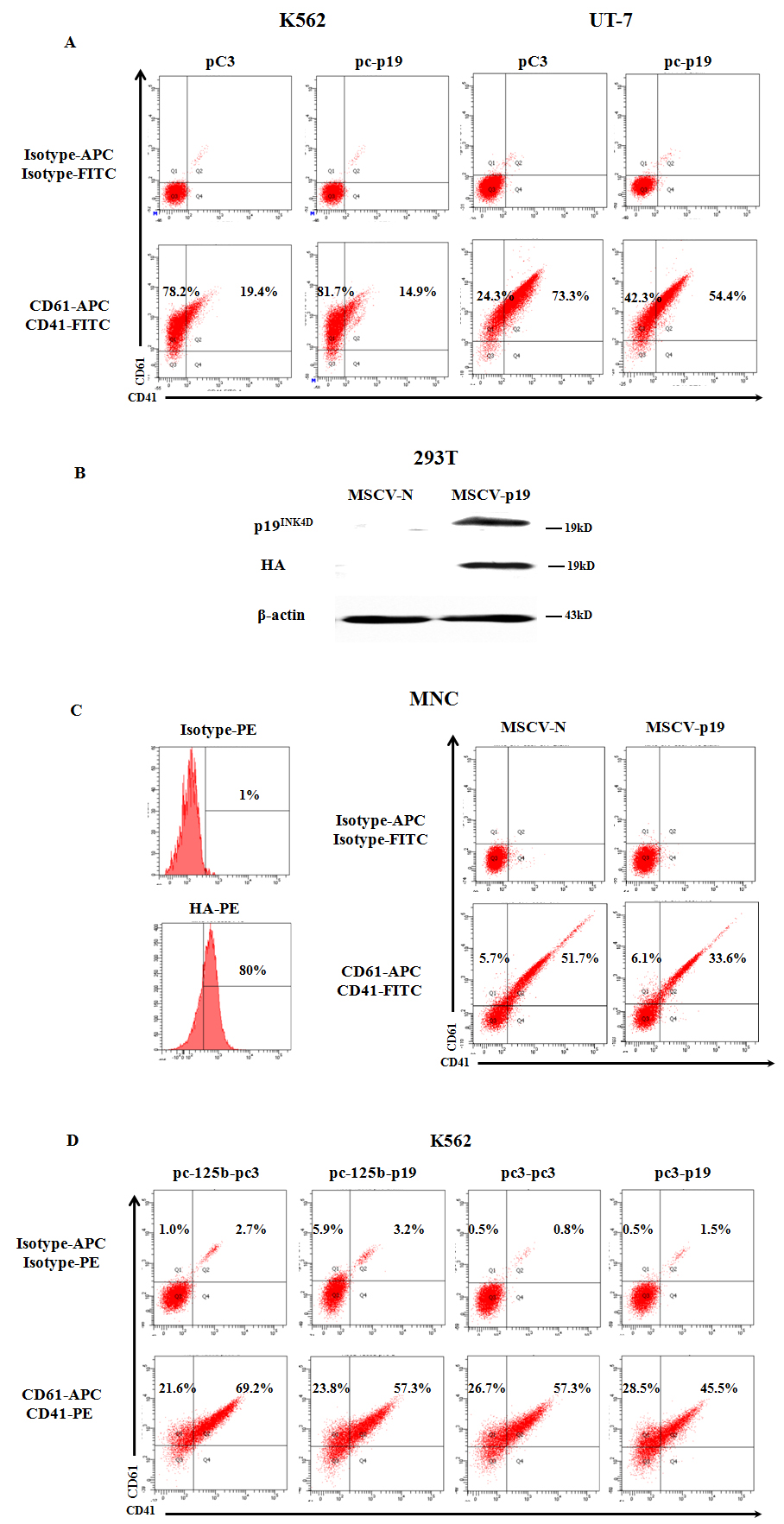
Figure S3.Representative flow cytometricplots demonstrate effects of p19^INK4D^ overexpression on megakaryocytic surface marker expression. (A) Impact of p19^INK4D^ overexpression on K562 and UT-7 cells. (B)293T cells were infected with retrovirus carrying HA-tagged p19^INK4D^(MSCV-p19)or control blank virus (MSCV-N). 3 days after infection, p19^INK4D^and HA tag expression was confirmed by western blot analysis.(C) Impact of p19^INK4D^ overexpression on cord blood mononuclear cells (MNCs). The infection efficiency was almost 80% confirmed by FACS examination. (D) Impact of p19^INK4D^ and miR-125b co-up-regulation on K562 cells.
